# How loneliness linked to anxiety and depression: a network analysis based on Chinese university students

**DOI:** 10.1186/s12889-023-17435-4

**Published:** 2023-12-13

**Authors:** Mengyuan Yang, Wenwen Wei, Lei Ren, Zhaojun Pu, Yuanbei Zhang, Yu Li, Xinhong Li, Shengjun Wu

**Affiliations:** 1 Department of Military Medical Psychology, Air Force Medical University, 169 West Changle Road, Xi’an, 710032 Shaanxi China; 2 Military Psychology Section, Logistics University of PAP, Tianjin, 300309 China; 3 Military Mental Health Services & Research Center, Tianjin, 300309 China; 4 Academic Affairs Office, Air Force Medical University, 169 West Changle Road, Xi’an, 710032 Shaanxi China; 5 Department of General Medicine, Tangdu Hospital, Xi’an, 712046 Shaanxi China

**Keywords:** Loneliness, Depression, Anxiety, Network analysis, University students, Cross-sectional study

## Abstract

**Background:**

There is conclusive evidence of a multifaceted and bidirectional relationship between loneliness and depression and anxiety. Nonetheless, more extensive research is needed to examine their relationships at a more granular level. This study employed a network analysis approach to identify the pathological mechanisms underpinning those relationships and to identify important bridge nodes as potential targets for intervention.

**Methods:**

941 University students were included in this study. The ULS-6 (the short-form UCLA Loneliness Scale) was used to assess loneliness, the PHQ-9 (Patient Health questionnaire-9) and GAD-7 (Generalized anxiety disorder 7-item) scales were used to assess the symptoms of depression and anxiety. We constructed two network structures of loneliness-anxiety and loneliness-depression and computed bridge expected influence for each symptom. In addition, we showed a flow network of “Suicide” containing symptoms of depression and loneliness.

**Results:**

All edges were positive in both networks constructed and the strongest edges were present within disorder communities. The overall connection between loneliness and depression was stronger compared to anxiety. The results demonstrated that the loneliness item “People are around me but not with me” was identified as bridge symptom in both networks. Furthermore, “Suicide” was directly connected to five symptoms of depression and four items of loneliness, with the strongest connections being between it and “Feeling of worthlessness” and “Psychomotor agitation/retardation”.

**Conclusions:**

Our findings provide a more nuanced explanation of the link between loneliness and depression and anxiety. The results identified the bridge symptom “People are around me but not with me”, which had the strongest effect on enhancing symptoms of depression and anxiety. Clinical improvements based on the findings of this study and the impact of the intervention are discussed.

**Supplementary Information:**

The online version contains supplementary material available at 10.1186/s12889-023-17435-4.

## Introduction

During the transition from late adolescence to adulthood, university students find themselves in a critical stage, which is typically accompanied by social, structural, and behavioral changes, making the mental health of university students a subject of great concern for schools and communities [1, 2]. Research has shown that high academic pressure [3], unhealthy competition [4], negative parenting styles [5], poor interpersonal relationships [6], and other factors can contribute to depression and anxiety among college students. Additionally, research has shown that smartphone addiction can worsen symptoms of anxiety and depression, resulting in sleep disturbances and reduced sleep quality [7]. A meta-analysis revealed a depression prevalence of 28.4% among Chinese university students [8], while the estimated prevalence of anxiety during the COVID-19 pandemic was 41% [9]. These symptoms of anxiety and depression can have negative effects on cognition and behavior, potentially influencing academic performance [10]. Furthermore, research has shown that prolonged periods of mild depression are significant risk factors for suicidal thoughts [11]. As the propensity for mental health problems can have serious adverse effects and behavioral consequences, continuous assessment of students’ mental health status, identification of important causal mechanisms, and the development of personalized treatment plans to address their individual needs are essential [2].

Loneliness has garnered significant attention in recent years due to its close association with anxiety and depression [12]. Loneliness is a state in which an individual subjectively experiences a quantitative or qualitative lack of intimate and social relationships [13]. Loneliness is not merely an individual condition but rather a recurring aspect of human social life, interwoven with developmental, evolutionary, and cultural roots [14]. Consequently, it is regarded as a global public health concern [14, 15]. Findings showed that loneliness increased with individualism, decreased with age, and was greater in men than in women [16]. Loneliness can be influenced by various socio-cultural contexts and may yield distinct effects and outcomes within diverse cultural frameworks. While previous research has predominantly focused on the risk factors and outcomes of loneliness in elderly individuals [17, 18], recent studies have demonstrated that loneliness is also prevalent among young individuals [19, 20]. Notably, a survey on community life discovered that young adults aged 16 to 24 are more susceptible to loneliness than older age groups [21]. Among university students, 32.4% reported moderate levels of loneliness, while 3.2% reported severe loneliness [12]. The COVID-19 pandemic has further highlighted the heightened risk of loneliness among young adults aged 18 to 30 [22]. Longitudinal studies have found a reciprocal relationship between loneliness and depression, with depression mediating the predictive effect of loneliness on anxiety over time [23]. Furthermore, within the adolescent depression network, suicidal thoughts exhibit the strongest direct association with loneliness and loneliness explained most variance of suicide ideation [24]. Research has also shown that loneliness is directly linked to suicide ideation even when controlling for depression [25]. Young individuals who experience loneliness are more vulnerable to mental health problems [26], engage in risky behaviors, adopt negative coping strategies, face an increased risk of unemployment [27], and are significantly linked to a higher overall risk of mortality [28, 29]. Given the intricate and bidirectional nature of the relationship between loneliness and mental illness, an understanding of the underlying mechanisms tying loneliness to common symptoms like depression and anxiety can inform the development of effective intervention strategies aimed at mitigating their adverse impact.

Network analysis, an increasingly employed data-driven analytical approach in psychopathology research, allows for the visual depiction of intricate relationships between variables in a network [30–32]. In contrast to traditional latent variables, network analysis conceptualizes psychopathological structures as interconnected symptom nodes, with edges denoting associations between two nodes while accounting for other variables in the network [33, 34]. According to this perspective, mental disorders emerge as a result of interactions between variables rather than stemming from a common underlying cause [33, 35]. The accurate depiction of these interactions is essential for understanding psychopathological mechanisms and developing targeted intervention strategies. Therefore, network analysis is a suitable method for exploring the complex connections between loneliness and depression and anxiety [31, 36]. Furthermore, when exploring the interaction among symptoms of mental disorders, network analysis offers valuable indices to assess the significance of nodes [37, 38]. It also provides bridge centrality index to assess the relative importance of a given node in increasing the risk of community transmission to other disorders communities within the network [37, 39]. Therefore, identifying bridging symptoms among distinct mental disorders is beneficial since it aids in tailoring treatment to specific problems, and suppressing them will hopefully halt the onset of other symptoms within the network [35, 40].

Previous studies have utilized network analysis methods to examine the intricate relationship between loneliness and anxiety and depression [31, 36, 41]. Grygiel’s study mainly investigated the role played by loneliness in the network of depression symptoms treating loneliness as a node within the network, the 11-item De Jong Gierveld Loneliness Scale and a single direct question were completed to evaluate loneliness in 496 Polish adolescents. The study’s results reveal that loneliness has the lowest level of centrality among all elements of the network and was not affected by the method of measurement, unlike in previous studies, which could be attributed to the differences in content and entry order included in the scale [31]. To address these limitations, Owczarek et al. constructed a network wherein different elements of loneliness, depression, and anxiety were treated as nodes [36]. However, the results did not support the notion that addressing loneliness effectively diminishes anxiety and depression. This outcome may stem from the items representing loneliness being tightly clustered and largely isolated from the rest of the network. Moreover, items from the loneliness cluster showed low bridge expected influence and lowest values of the closeness, along with the results from the Clique Percolation. In brief, these results could suggest that there exist constructs that act as bridges between loneliness and the other two constructs that have not been captured by the present study [36, 37]. Additionally, recent network analysis studies on depression have identified loneliness as the most central node, signifying its strongest association with other symptoms of depression [24, 42]. Thus, further research is needed to investigate the complex relationship between loneliness and depression and anxiety, providing more concrete evidence to guide clinical interventions and improvements. Furthermore, network analysis can help us better understand how symptoms are related to suicidal ideation. This suggests that investigating the connection between suicidal thoughts and symptoms of depression and loneliness from a network analysis perspective may provide valuable evidence for early prevention and clinical intervention in addressing suicide among university students [43, 44].

The aim of this study is to use network analysis method to investigate the association between loneliness and symptoms of anxiety and depression among Chinese university students. Separate network models will be constructed for loneliness and depression, as well as loneliness and anxiety, to explore potential connections between these symptoms within a sample of Chinese university students. Particular attention will be focused on identifying bridge symptoms to assess the interconnectivity of symptoms in the two constructed networks. Particularly, we focus on the symptoms that are directly related to “thoughts of death”. This is an exploratory study with no specific hypotheses formulated.

## Methods

### Participants and procedures

Our study collected data from university students in Xi’an city using the snowball method to distribute online questionnaires from 10th February 2023 to 26th February. We distributed the questionnaires through WeChat as it has the broadest user base in China and is utilized by nearly all university students. We first randomly selected some college students to take the questionnaire and hoped that they would forward the questionnaire to invite more students to participate in this study. Inclusion criteria included (1) currently enrolled university students; (2) not having a self-reported history of neurological or psychiatric disorders; (3) consent to volunteer for this study. 84 questionnaires were excluded according to two attention check items and response time and 11 questionnaires were excluded due to incomplete basic information or incomplete questionnaire content. A total of 941 questionnaires were eventually included.

### Ethics considerations

Ethical approval was obtained from the First Affiliated Hospital of the Fourth Military Medical University. In the first part of the questionnaire. we informed each participant of the purpose of the study and also provided one consent form with the ethical guidelines addressing the need. If participants agree to take part, they should click “I agree” to continue with the following project. Participants are also informed that the interests of our research subjects will be protected at any time.

### Measures

#### Depression symptoms

The Patient Health Questionnaire-9 (PHQ-9) is a 9-item self-report measure examining the presence and severity of depression symptoms over the past 2 weeks according to the Diagnostic and Statistical Manual of Mental Disorders IV (DSM-IV) criteria [45]. The score of each item ranges from 0 (not at all) to 3 (nearly every day), with a summed score ranging from 0 to 27. Higher scores indicate more severe depressive symptoms. The Chinese version of the PHQ-9 has been shown to be an effective screening tool for depression [46]. The scale demonstrated good internal consistency in the current study (Cronbach’s α = 0.900).

#### Anxiety symptoms

The Generalized Anxiety Disorder 7-Item Questionnaire (GAD-7) is a 7-item self-report measure examining the presence and severity during the past two weeks [47]. The score of each item ranged from 0 (not at all) to 3 (nearly every day) and the GAD-7 scale score ranges from 0 to 21. Higher scores indicate more severe anxiety symptoms. The Chinese version of the GAD-7 has been validated and is widely used [48, 49]. The scale demonstrated excellent internal consistency in the current study (Cronbach’s α = 0.931).

#### Loneliness symptoms

The short-form UCLA Loneliness Scale (ULS-6) is a 6-item scale with a 4-level frequency score, ranging from 1 to 4 (point referred to “never”, “rarely”, “sometimes” and “always”, respectively). The total score ranges from 6 to 24, with higher scores indicating a greater degree of loneliness. The ULS-6 is an appropriate measure for the cross-cultural measurement and comparison of loneliness [50] and the Chinese version of ULS-6 demonstrated excellent psychometric properties [51, 52]. The scale demonstrated excellent internal consistency in the current study (Cronbach’s α = 0.925).

### Network analysis

#### Network structure

Data were sorted with Excel software, and data analysis was performed using R software (version 4.2.1) and its software package. The network was estimated via Gaussian graphical model [53]. Within the network, edges represent the partial correlation between two nodes while adjusting for the effects of all the other nodes. To account for the ordinal nature of the PHQ-9, GAD-7 and ULS-6, we estimated regularized partial correlation networks via the Extended Bayesian Information Criterion (EBIC) Graphical LASSO, as recommended by Epskamp and Fried [54]. By shrinking all edges and punishing the edges of trivially small partial correlation coefficients to zero, this regularization process assisted in removing false connections and obtaining a more robust, sparse, and understandable network [54, 55]. The Extended Bayesian Information Criterion (EBIC) hyperparameter γ was set to 0.5 to balance sensitivity and specificity [56, 57]. The layout of the presented networks was based on the Fruchterman-Reingold algorithm [58]. In the presented network, blue edge represents positive correlation and red edge represents negative correlation, the strength of the correlation is reflected by the thickness of the edge. The R-package “qgraph” (version 1.9.2) was used to compute the presented network [34].

We utilized the “flow” graphical function in the R-package qgraph to ascertain which symptoms are directly linked to the depressive symptom of “suicide” in the depression-loneliness network. This function arranges “suicide” on the left and constructs a vertical network to show the direct or indirect association with it.

#### Bridging symptoms

In order to identify the bridge symptoms, bridge expected influence (BEI) was calculated. BEI measures the sum of the value of all edges connecting a given node to all nodes in the other communities. Compared to traditional bridge centrality indices, bridge expected influence is a better approach for identifying bridge nodes in the network containing positive and negative edges [37]. Higher BEI values suggest a higher likelihood of increasing the risk of contagion to other communities [37, 59]. Based on previous studies, we employed a rigorous method to identify bridge symptoms by applying a 90th percentile blind cut-off to the expected BEI score to avoid possible confirmation bias [43]. The bridge expected influence was calculated via the R package networktools (version 1.5.0) [37].

#### Network stability and accuracy

First, we evaluated the accuracy of edge weights by plotting the 95% confidence interval (with 2,000 bootstrap samples) for each edge within the presented networks. A narrower 95% confidence interval indicates a more reliable network [43, 60]. Second, we computed the correlation stability (CS) coefficient to evaluate the stability of node bridge expected influence using a case-dropping bootstrap method (with 2,000 bootstrap samples). CS coefficient represents the maximum percentage of sample cases that can be dropped from the original full cases to retain a correlation of 0.7 in at least 95% of the samples. CS coefficients should be higher than 0.25 (acceptable level), preferably higher than 0.5 [60]. Finally, we further conducted bootstrapped difference tests (with 2,000 bootstrap samples) for edge weights and bridge expected influence. The aforementioned procedures were conducted via the R-package bootnet (version 1.5) [60].

## Results

### Demographic characteristics

The mean age of the 941 university students was 20.69 ± 1.88 years (M ± SD, range17-25 years), and the majority of them were male (n = 880, 93.52%). Of all participants, 378 were only children (40.17%) and 91 were single parents (9.67%), 333 individuals with a monthly household income of 5,000 or higher per capita (35.39%). Participants’ ULS-6, PHQ-9 and GAD-7 scores reflects the full range of symptom severity. Given that the participants were non-clinical patients, 267 of all participants had mild to severe symptoms of depression (28.37%) and 155 had mild to severe symptoms of anxiety (16.47%). Table 1 shows abbreviation, mean scores and standard deviations for each symptom items in the present networks.

**Table 1:** EI, BEI values and abbreviation for each node of the ULS-6, PHQ-9 and GAD-7

Nodes	Abbreviation	M	SD
**UCLA Loneliness Scale(ULS-6)**			
ULS1: Lack companionship	Companion	1.72	0.78
ULS2: No one I can turn to	Seek help	1.53	0.68
ULS3: Feeling left out	Left out	1.45	0.64
ULS4: Feeling isolation from others	Isolation	1.49	0.64
ULS5: Unhappy being so withdrawn	Unhappy	1.39	0.61
ULS6: People are around me but not with me	With me	1.38	0.60
**Depression symptoms (PHQ-9)**			
PHQ1: Anhedonia	Anhedonia	0.52	0.66
PHQ2: Depressed or sad mood	Sad mood	0.38	0.59
PHQ3: Sleep difficulties	Sleep	0.44	0.67
PHQ4: Fatigue	Fatigue	0.50	0.66
PHQ5: Appetite changes	Appetite	0.38	0.67
PHQ6: Feeling of worthlessness	Worthless	0.30	0.58
PHQ7: Concentration difficulties	Concentration	0.42	0.68
PHQ8: Psychomotor agitation/retardation	Psychomotor	0.22	0.49
PHQ9: Thoughts of death	Suicide	0.11	0.36
**Anxiety symptoms (GAD-7)**			
GAD1: Nervousness or anxiety	Nervous	0.33	0.57
GAD2: Uncontrollable worry	Control worry	0.32	0.60
GAD3: Worry too much	Too much worry	0.29	0.57
GAD4: Trouble relaxing	Relax	0.23	0.52
GAD5: Restlessness	Restless	0.18	0.47
GAD6: Irritable	Irritable	0.20	0.46
GAD7: Afraid something will happen	Afraid	0.20	0.47

Abbreviations: M mean, SD Standard deviation

### Network structure

#### The loneliness-anxiety network

**Fig. 1:**
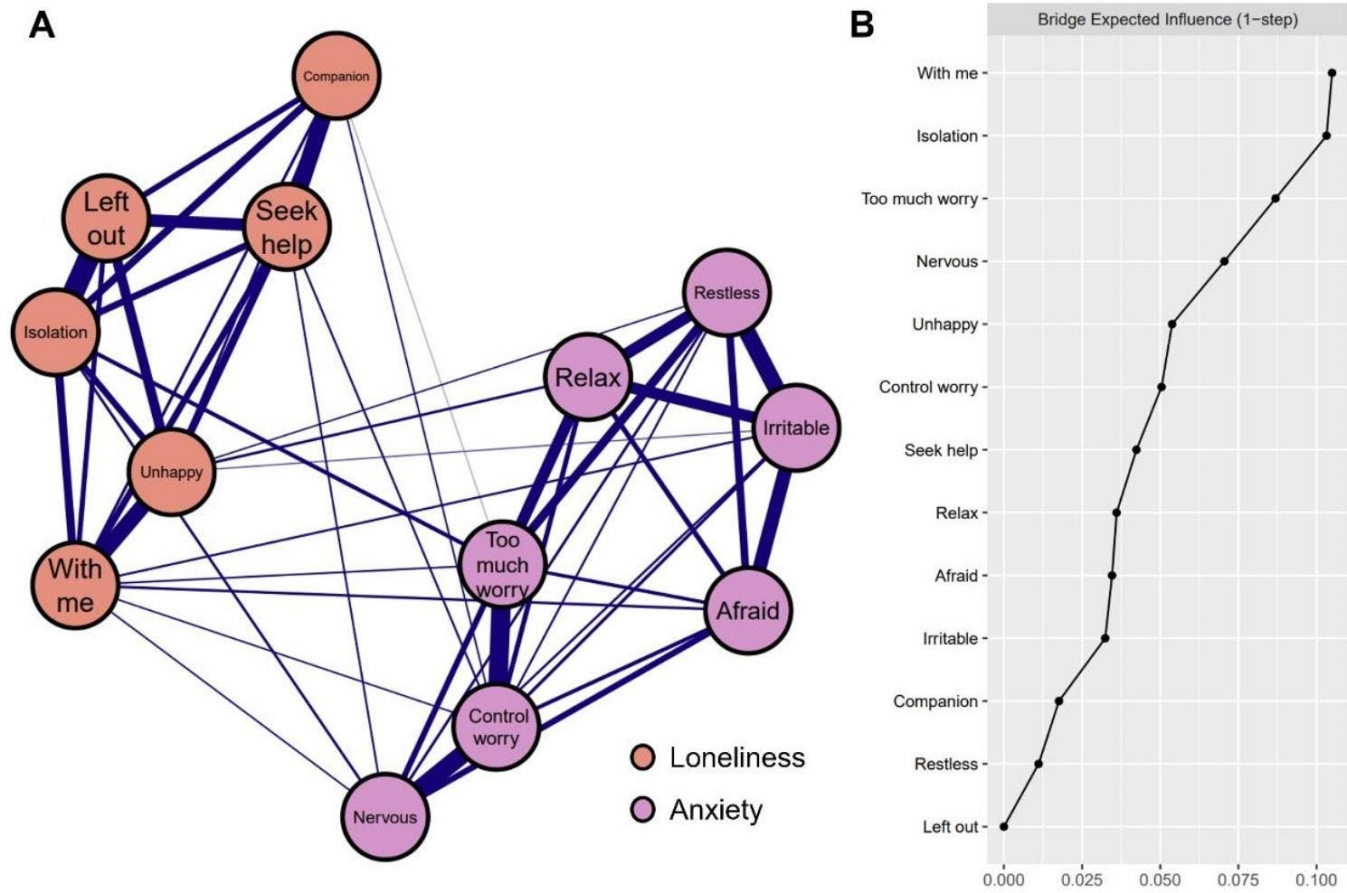
Network structure of loneliness-anxiety network and bridge expected influence for each node. (**A**) Blue edges represent positive correlations. The thickness of the edge reflects the magnitude of the correlation (cut = 0.01, layout = “spring”) (**B**) The BEI of each node in the network (raw-value)

The Loneliness-anxiety network is depicted in Fig. 1A and exhibits the subsequent traits. First, 49 edges are not zero (62.8%) among 78 possible edges and all these edges are positive. Second, six strongest edges were identified in the final network. Three of the strongest edges were between the loneliness symptoms “Left out” and “Isolation” (weight = 0.45), “Companion” and “Seek help” (weight = 0.34) and between “Unhappy” and “With me” (weight = 0.32). The other three strongest edges existed within the anxiety community, which were between “Control worry” and “Too much worry” (weight = 0.40), “Nervous” and “Control worry” (weight = 0.38) and between “Restless” and “Irritable” (weight = 0.35). Third, thirteen cross-community edges were discovered in the network, which were weaker than the intra-community edges. We found “With me” had the more connections with symptoms of anxiety than other components of loneliness, which was linked to anxiety symptoms “Nervous”, “Control worry”, “Too much worry”, “Irritable” and “Afraid” (weight = 0.01, 0.01, 0.02, 0.03 and 0.03, respectively). The strength of the edge between “isolation” and “Too much worry” (weight = 0.07) was larger than that of any other cross-community edge. All the edge weights within the loneliness-anxiety network were presented in Supplementary Table S1. Bootstrapped 95% confidence intervals for estimated edge weights were relatively narrow indicating that edge weights were relatively reliable (Fig. S1 in the supplementary material). In addition, the result of bootstrapped difference test for edge weights indicates that the six strongest edge weights are significantly higher than about 80–94% proportion of the other edge weights (Fig. S2 in the supplementary material).

The bridge expected influence of anxiety and loneliness symptoms are shown in Fig. 1B. Two loneliness items “With me” (BEI = 0.11) and “Isolation” (BEI = 0.10) were identified as bridge symptoms, indicating that they have the strongest capability to increase the risk of contagion to anxiety in the existing network. The correlation stability coefficient of node bridge expected influence was 0.44, above the recommended threshold of 0.25, suggesting that bridge expected influence estimation had an acceptable level of stability. (Fig. S3 in the supplementary material). Furthermore, the bootstrapped difference test for node bridge expected influences showed that the bridge expected influence of node “With me” was significantly higher than 50% of the other nodes in the current network (Fig. S4 in the supplementary material).

#### The loneliness-depression network

**Fig. 2:**
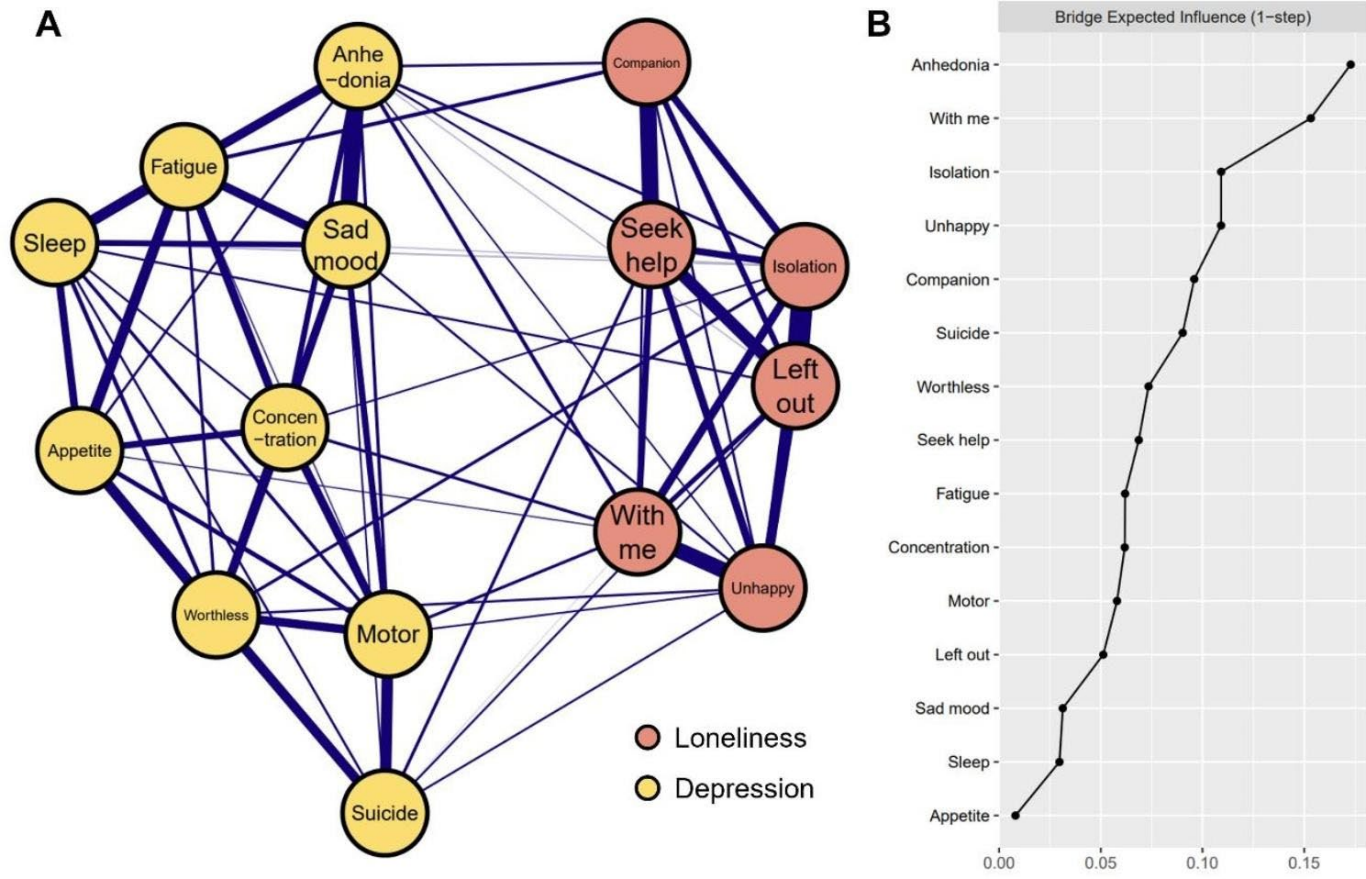
Network structure of loneliness-depression network and bridge expected influence for each node. (**A**) Blue edges represent positive correlations. The thickness of the edge reflects the magnitude of the correlation (cut = 0.01, layout = “spring”) (**B**) The BEI of each node in the network (raw-value)

The Loneliness-depression network is shown in Fig. 2A and it has the following characteristics. First, 68 edges are not zero (64.8%) among 105 possible edges and all these edges are positive. Second, four strongest edges were identified in the final network. Three of the strongest edges were between the loneliness symptoms “Left out” and “Isolation” (weight = 0.45), “Companion” and “Seek help” (weight = 0.34) and between “Unhappy” and “With me” (weight = 0.32). The strongest edge in the depression community was between “Anhedonia” and “Sad mood” (weight = 0.34). Third, eighteen cross-community edges were discovered in the network, which were weaker than the intra-community edges. We found “Unhappy” had more connections with symptoms of depression than other components of loneliness, which was linked to depression symptoms “Anhedonia”, “Sad mood”, “worthless”, “Motor” and “Suicide” (weight = 0.01, 0.03, 0.03, 0.02 and 0.02, respectively). Two strongest cross-community edges were between “Anhedonia” and “With me” (weight = 0.06), and between” Fatigue” and “Lack companionship” (weight = 0.06). Supplementary Table S2 presents all the edge weights within the Loneliness-depression network. Bootstrapped 95% confidence intervals for estimated edge weights were relatively narrow indicating that edge weights were relatively reliable and accurate (Fig. S5 in the supplementary material). Moreover, in the current network, bootstrapped difference test for edge weights indicates that the four strongest edge weights are significantly higher than about 88–100% proportion of the other edge weights (Fig. S6 in the supplementary material).

The bridge expected influences of depression and loneliness symptoms are shown in Fig. 2B. Based on the demonstrated results, depression item “Anhedonia” (BEI = 0.17) and loneliness item “With me” (BEI = 0.15) were identified as bridge symptoms. This indicates that “Anhedonia” has the strongest capability to increase the risk of contagion to loneliness, and “With me” has the strongest capability to increase the risk of contagion to depression in the existing network. The correlation stability coefficient of node bridge expected influence is 0.52, suggesting that the estimates for the expected influence of the node bridge are sufficiently stable (Fig. S7 in the supplementary material). Furthermore, bootstrapped difference tests for node bridge expected influence show that the bridge expected influence of bridge symptoms are significantly higher than about 60% proportion of the other symptoms (Fig. S8 in the supplementary material).

**Fig. 3:**
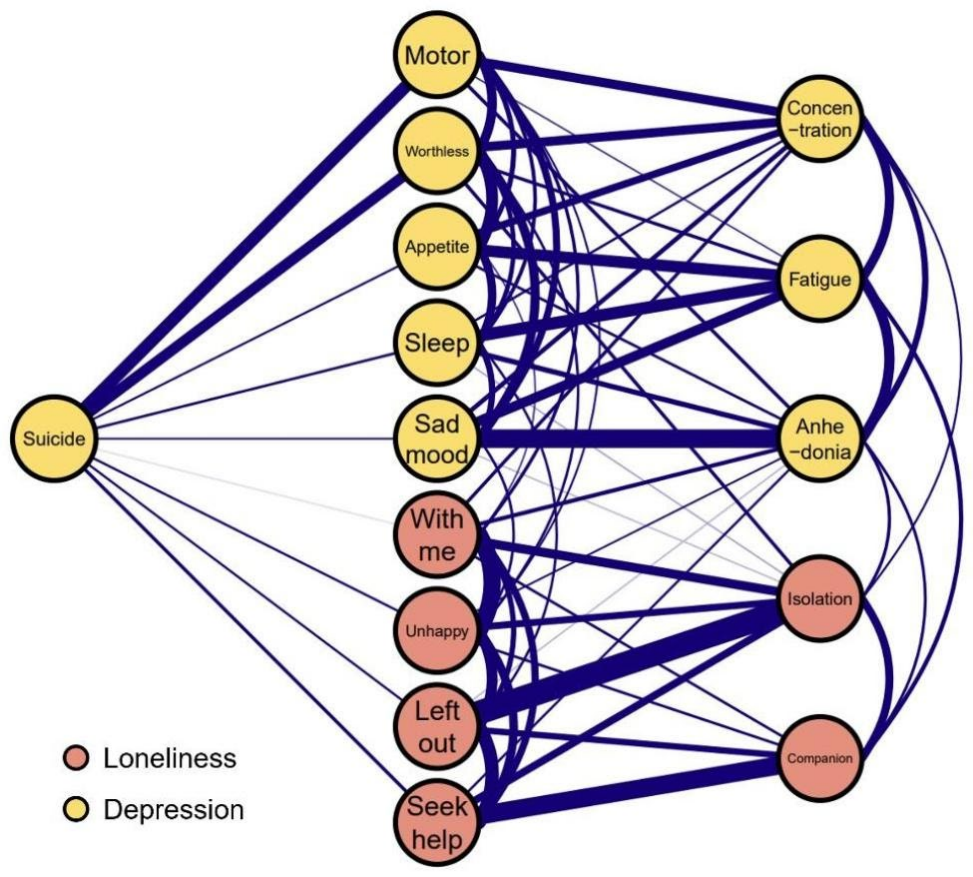
Flow network of suicide thoughts. Blue edges represent positive correlations, the thickness of the edge reflects the magnitude of the correlation

Figure 3 depicts a flow diagram showing how depression symptom “suicide” is connected to all other symptoms of the loneliness-depression network. The result indicates that nine symptoms are directly linked to “suicide” and five symptoms are not directly linked to “suicide”. The direct relations between “suicide” and depression symptoms “psychomotor agitation/retardation” (weight = 0.22) and “feeling of worthlessness” (weight = 0.19) are the strongest direct relations.

## Discussion

The present study provided network structures to revealed how loneliness linked to depression and anxiety at the symptom level in a population of Chinese university students. Within the two networks constructed, it was observed that the strongest edges were present within respective disorders. In comparison, edges between different communities were weaker than those within communities, which is consistent with previous findings [36, 41]. In the study, we observed that the strongest cross-community edges were between “Anhedonia” and “With me”, between “Fatigue” and “Lack companionship” and between “isolation” and “Too much worry”. Even though significantly weaker than the intra-community edges, the findings identify aspects of loneliness that may be most closely associated with the presence of underlying psychopathology for symptoms of depression and anxiety in this population. These candidates may warrant further longitudinal studies to more rigorously investigate their potential role in exacerbating depression and anxiety in psychopathology.

Overall, various aspects of loneliness had distinct associations with symptoms of depression and anxiety. Although stable connections existed between loneliness and both depression and anxiety, loneliness and depression showed a stronger relationship, given that the mean values of BEI for the nodes of depressive symptoms in the loneliness-depression network were higher than those of the nodes of anxiety symptoms in the loneliness-anxiety network. Additionally, the values of BEI for the nodes of different aspects of loneliness were also higher, consistent with previous studies [41]. In a study including different symptoms of cognitive, affective and physical, loneliness was identified as one of the most central symptoms in the depression network and the sadness-loneliness edge was identified as one of the strongest associations in the network [42]. However, the affective aspect of depressive symptoms, “sadness”, had a relatively low BEI in our study. This may be attributable to discrepancies in the study population and measurement tools [42]. In the present study, “Anhedonia” has the highest degree of BEI, which implies that an intervention targeting “Anhedonia” would have the most significant impact on alleviating symptoms of loneliness in the population of Chinese university students [37]. “Anhedonia” constitutes one of the two key symptoms of a major depressive episode, and dysfunction in any component of reward processing can lead to anhedonia, including reward learning, appraisal, motivation and effort expenditure [61]. Studies have shown that the prevalence of loneliness was 93.8% in participants with depressive psychosis, loneliness was also significantly associated with anhedonia [62]. Moreover, anhedonia is a crucial element for the association between quality of life, sleep problems, and negative cognitions [63]. Additionally, it has been discovered that individuals exhibiting social anhedonia (SA) may experience diminished social connectedness and feelings of loneliness, and loneliness served as a complete mediator of the link between SA and general social functioning [64]. This also indicates that interventions aimed at the two bridge nodes of “Anhedonia” in depression and “With me” in loneliness may have a better impact on reducing the whole network of depressive and loneliness symptoms and thus improving the overall social functioning of university students, which needs to be verified in further clinical interventions.

Based on the results of bridge centrality, “People are around me but not with me” was identified as bridge symptom in both networks. The result demonstrated that when loneliness is present, interventions focused on “People are around me but not with me” may decrease the risk of developing depression and anxiety. Research has highlighted loneliness as a unique condition in which an individual perceives himself or herself to be socially isolated, even when surrounded by others [15]. The bridge symptom we identified implies a sense of not being cared for or belonging to a group, which differed from the previous findings of Rodriguez et al. [41]. This could be attributable to differences in cultural contexts. While loneliness is typically associated with individualistic, in the Chinese culture context, which tends to prioritize collectivism, the deficiency of togetherness and consideration from the group may have a more profound effect on individuals [16]. Despite the significance of social networks for mental health and well-being, however, research has revealed that the correlation between loneliness and the size of the social network and the frequency of interaction with others is weak [65]. Individuals can reside alone and not experience loneliness, or they can be encompassed by others and still encounter loneliness [66]. Thus, the key factor for maintaining mental health appears to be the subjective perception of social support rather than the amount of support available to the individual or the objective nature of the real world [67], which corroborates the results of our study. Recent research suggests that interventions aimed at reducing potential perceptions of loneliness through cognitive behavioral therapy (CBT), such as reappraising solitude to recognize the benefits of loneliness, or improving social skills to enhance social support, may be effective [68, 69]. And addressing poor social cognition, instructing individuals to identify their automatic negative thoughts, false expectations and attributions about others and social interactions, is considered the most potentially valuable intervention strategy among the different types available [68]. Increasingly, community projects, behavioral interventions, and online resources should be utilized to address loneliness.

In the constructed suicidal flow network, “Suicide” displayed the strongest direct associations with “Psychomotor agitation/retardation” and “Feeling of worthless”, aligned with prior research [43, 70]. In a study of patients with major depressive episode (MDE), psychomotor agitation and impulsivity was the variable most frequently associated with previous suicide attempts [71], it is therefore proposed that early recognition of symptoms such as psychomotor agitation and impulsivity in patients with MDE may be an important step in suicide prevention. Moreover, among depressive symptoms during an MDE, “feelings of worthlessness” is the only significant indicator of elevated risk of suicide attempt after the episode has remitted, beyond previous suicide attempts [72]. In a recent study of clinically stable adolescents with the recurrent depressive disorder during the COVID-19 pandemic, suicidal ideation had the strongest direct correlation with the PHQ6 “guilty” [70]. Nonetheless, the link between suicidal ideation and “guilt” is supported by other evidence that increased negative self-referential thinking and negative beliefs about the self are potential risk factors for suicide [73, 74]. In addition to this, “Suicide” is directly linked to several specific symptoms of loneliness, such as “Unhappy being so withdrawn”, “Left out” and “Seek help”. The strongest connection was found between “Suicide” and “Seek help”, which has not been reported in previous study. It indicates that interventions targeting “No one I can turn to” can effectively decrease suicidal ideation, implying that the availability of support, or social support, is a crucial contributor to suicidal ideation. In addition, previous research has demonstrated that social support is associated with lower risk factors for suicide and that it is important to consider social support when discussing disclosure of suicide attempts [75]. Furthermore, social support has a direct protective effect against suicidal ideation [65]. Combining these findings, our study suggests that early identification and intervention for “Psychomotor agitation/retardation” and “worthlessness” and improving the level and amount of social support are crucial in reducing suicidal ideation among Chinese university students. It is critical to consider the level and amount of social support to reduce suicidal ideation.

To the best of our knowledge, this is the first study of the network structure between loneliness and depression and anxiety in a group of Chinese university students. The present study yields potential implications for clinical intervention and prevention strategies aimed towards catering to the mental health needs of Chinese university students. Firstly, the findings identified a number of unique associations between different symptoms of depression and anxiety and different aspects of loneliness, and found that the overall connection between loneliness and depression was stronger compared to anxiety. From a network perspective, severing or attenuating specific connections between different associations may have implications for improvements in co-morbid symptoms. Secondly, “People are around me but not with me” was identified as a bridging symptom in both the loneliness-depression network and the loneliness-anxiety network. Therefore, the possibility of developing depression or anxiety is higher when surrounded by people but lacking care. Interventions that target bridging nodes have been found to be more effective in reducing the risk of transmission. However, due to the cross-sectional nature of the current study, it is not feasible to ascertain whether the most prominent node is indeed the causal factor of the other symptoms or merely a consequential outcome within the network. Thirdly, there is a strong association between “suicide” and depression symptoms “psychomotor agitation/retardation”, and “feelings of worthlessness” and a direct correlation between the specific aspect of loneliness “No one I can turn to”. By observing and addressing the aforementioned symptoms in Chinese university students, it may be possible to efficiently detect and intervene in cases of suicidal ideation.

There are several limitations need to be considered when interpreting our results. Firstly, the participants in the present study were university students and mostly male. While certain studies have indicated that there are no differences in the association between loneliness and physical and mental health across gender groups [76, 77], this does not mean that their network structures are consistent, which may limit the generalizability of our findings. It would therefore be valuable to conduct comparative research on network structures between genders in the future. The second limitation concerns the use of a cross-sectional research design. The cross-sectional design hinders the identification of causal or temporal relationships between different symptoms, so the results of this study should be further explored and validated with longitudinal data. Third, there were nodes in our study that were closely related to each other, and they may have measured the same constructs or had significant overlap due to the wording of the items and response options. Therefore, these associations are not emphasized in the current findings. Fourth, the network structure constructed here investigates between-subject effects on a group level. This implies that the network structure may not be replicated in the same way within a single individual. Ultimately, the results of this study provide a specific target for clinical intervention while additional intervention studies are needed to further confirm and extend our findings.

## Conclusion

In conclusion, this study is the first article investigating the network structure between loneliness and depression and anxiety in a group of Chinese university students. The results of BEI revealed that “Anhedonia” and “With me” were identified as bridge symptoms. Furthermore, “With me” was also identified as a bridge symptom in the loneliness-anxiety network. This suggests that interventions targeting “Anhedonia” may be useful in improving symptoms of loneliness, and that among university students with feelings of loneliness, focusing on and improving “With me” may be important in preventing and reducing the onset of depression and anxiety. Results also identify depression symptoms “Psychomotor agitation/retardation” and “Feeling of worthlessness”, as well as “Seek help” of loneliness as critical priorities due to they are their association with “Suicide”. The clinical implications of treating specific symptoms as a goal of prevention and intervention are discussed.

### Electronic supplementary material

Below is the link to the electronic supplementary material.

Supplementary Material 1

Supplementary Material 2

## Data Availability

The datasets used and/or analyzed during the current study are available from the corresponding author on reasonable request.

## Abbreviations

ULS-6: The short-form UCLA Loneliness Scale
PHQ-9: Patient Health Questionnaire-9
GAD-7: Generalized Anxiety Disorder 7-Item Questionnaire
DSM-IV: Diagnostic and Statistical Manual of Mental Disorders IV
LASSO: Least absolute shrinkage and selection operator
EBIC: Extended Bayesian Information Criterion
M: Mean
SD: Standard deviation
BEI: Bridge expected influence
SA: Social anhedonia
MDE: Major Depressive Episode
