# Supplementary material for: How loneliness linked to anxiety and depression: a network analysis based on Chinese university students
Source: BMC Public Health. 2023 Dec 13;23:2499. doi: 10.1186/s12889-023-17435-4 (PMC10720215; doi:10.1186/s12889-023-17435-4)
Supplement: Supplementary file 1 — Supplementary Material 1 [file 12889_2023_17435_MOESM1_ESM.docx]

**How Loneliness Linked to Anxiety and Depression: A network analysis based on Chinese university students**

Mengyuan Yang^1^, Wenwen Wei^1^, Lei Ren^2,3^, Zhaojun Pu^1^, Yuanbei Zhang^1^, Yu Li^4^, Xinhong Li^5,*^, Shengjun Wu^1,*^

^1^Department of Military Medical Psychology, Air Force Medical University, 169 West Changle Road, Xi’an, Shaanxi 710032, China

^2^Military Psychology Section, Logistics University of PAP, Tianjin, 300309

^3^Military Mental Health Services & Research Center, Tianjin, 300309

^4^Academic Affairs Office, Air Force Medical University, 169 West Changle Road, Xi’an, Shaanxi 710032, China

^5^Department of General Medicine, Tangdu Hospital, Xi’an, Shaanxi 712046, China

^*^Correspondence: lxhfmmu@yahoo.com.cn; [wushj@fmmu.edu.cn](mailto:wushj@fmmu.edu.cn)

^†^ Mengyuan Yang and Wenwen Wei contributed equally to this work

**Supplementary Materials**

1. Table S1. Nonparametric Spearman rho correlation matrix of the loneliness and anxiety items
2. Table S2. Nonparametric Spearman rho correlation matrix of the loneliness and depression items
3. Figure S1. Accuracy of edge weights in the loneliness-anxiety network
4. Figure S2. Bootstrapped difference test for edge weights in the loneliness-anxiety network
5. Figure S3. Stability of node bridge expected influences in the loneliness-anxiety network
6. Figure S4. Bootstrapped difference test for node bridge expected influences in the loneliness-anxiety network
7. Figure S5. Accuracy of edge weights in the loneliness-depression network
8. Figure S6. Bootstrapped difference test for edge weights within the loneliness- depression network
9. Figure S7. Stability of node bridge expected influences in the loneliness- depression network
10. Figure S8. Bootstrapped difference test for node bridge expected influences in the loneliness-depression network

Table S1. Nonparametric Spearman rho correlation matrix of the loneliness and anxiety items

|  | **ULS1** | **ULS2** | **ULS3** | **ULS4** | **ULS5** | **ULS6** | **GAD1** | **GAD2** | **GAD3** | **GAD4** | **GAD5** | **GAD6** | **GAD7** |
| --- | --- | --- | --- | --- | --- | --- | --- | --- | --- | --- | --- | --- | --- |
| **ULS1** | 0 | 0.34 | 0.11 | 0.14 | 0.04 | 0.04 | 0 | 0.01 | 0 | 0 | 0 | 0 | 0 |
| **ULS2** | 0.34 | 0 | 0.25 | 0.13 | 0.15 | 0.12 | 0.02 | 0.02 | 0 | 0 | 0 | 0 | 0 |
| **ULS3** | 0.11 | 0.25 | 0 | 0.45 | 0.19 | 0.09 | 0 | 0 | 0 | 0 | 0 | 0 | 0 |
| **ULS4** | 0.14 | 0.13 | 0.45 | 0 | 0.12 | 0.16 | 0.04 | 0 | 0.07 | 0 | 0 | 0 | 0 |
| **ULS5** | 0.04 | 0.15 | 0.19 | 0.12 | 0 | 0.32 | 0 | 0 | 0 | 0.04 | 0.01 | 0.01 | 0 |
| **ULS6** | 0.04 | 0.12 | 0.09 | 0.16 | 0.32 | 0 | 0.01 | 0.01 | 0.02 | 0 | 0 | 0.03 | 0.03 |
| **GAD1** | 0 | 0.02 | 0 | 0.04 | 0 | 0.01 | 0 | 0.38 | 0.11 | 0.04 | 0.04 | 0.03 | 0.11 |
| **GAD2** | 0.01 | 0.02 | 0 | 0 | 0 | 0.01 | 0.38 | 0 | 0.4 | 0.09 | 0.02 | 0.05 | 0.06 |
| **GAD3** | 0 | 0 | 0 | 0.07 | 0 | 0.02 | 0.11 | 0.4 | 0 | 0.23 | 0.17 | 0 | 0.05 |
| **GAD4** | 0 | 0 | 0 | 0 | 0.04 | 0 | 0.04 | 0.09 | 0.23 | 0 | 0.23 | 0.23 | 0.09 |
| **GAD5** | 0 | 0 | 0 | 0 | 0.01 | 0 | 0.04 | 0.02 | 0.17 | 0.23 | 0 | 0.35 | 0.14 |
| **GAD6** | 0 | 0 | 0 | 0 | 0.01 | 0.03 | 0.03 | 0.05 | 0 | 0.23 | 0.35 | 0 | 0.28 |
| **GAD7** | 0 | 0 | 0 | 0 | 0 | 0.03 | 0.11 | 0.06 | 0.05 | 0.09 | 0.14 | 0.28 | 0 |

Table S2. Nonparametric Spearman rho correlation matrix of the loneliness and depression items

|  | **ULS1** | **ULS2** | **ULS3** | **ULS4** | **ULS5** | **ULS6** | **PHQ1** | **PHQ2** | **PHQ3** | **PHQ4** | **PHQ5** | **PHQ6** | **PHQ7** | **PHQ8** | **PHQ9** |
| --- | --- | --- | --- | --- | --- | --- | --- | --- | --- | --- | --- | --- | --- | --- | --- |
| **ULS1** | 0 | 0.34 | 0.1 | 0.14 | 0.03 | 0.03 | 0.03 | 0 | 0 | 0.06 | 0 | 0 | 0 | 0 | 0 |
| **ULS2** | 0.34 | 0 | 0.24 | 0.13 | 0.15 | 0.12 | 0.02 | 0 | 0 | 0 | 0 | 0 | 0 | 0 | 0.04 |
| **ULS3** | 0.1 | 0.24 | 0 | 0.45 | 0.19 | 0.08 | 0 | 0 | 0.03 | 0 | 0 | 0 | 0 | 0 | 0.02 |
| **ULS4** | 0.14 | 0.13 | 0.45 | 0 | 0.11 | 0.16 | 0.04 | 0 | 0 | 0 | 0 | 0.05 | 0.02 | 0 | 0 |
| **ULS5** | 0.03 | 0.15 | 0.19 | 0.11 | 0 | 0.32 | 0.01 | 0.03 | 0 | 0 | 0 | 0.03 | 0 | 0.02 | 0.02 |
| **ULS6** | 0.03 | 0.12 | 0.08 | 0.16 | 0.32 | 0 | 0.06 | 0 | 0 | 0 | 0.01 | 0 | 0.04 | 0.04 | 0 |
| **PHQ1** | 0.03 | 0.02 | 0 | 0.04 | 0.01 | 0.06 | 0 | 0.34 | 0.08 | 0.18 | 0.03 | 0 | 0.1 | 0.05 | 0 |
| **PHQ2** | 0 | 0 | 0 | 0 | 0.03 | 0 | 0.34 | 0 | 0.1 | 0.17 | 0 | 0.16 | 0.07 | 0.12 | 0.02 |
| **PHQ3** | 0 | 0 | 0.03 | 0 | 0 | 0 | 0.08 | 0.1 | 0 | 0.22 | 0.14 | 0.07 | 0.03 | 0.05 | 0.03 |
| **PHQ4** | 0.06 | 0 | 0 | 0 | 0 | 0 | 0.18 | 0.17 | 0.22 | 0 | 0.21 | 0.04 | 0.16 | 0.01 | 0 |
| **PHQ5** | 0 | 0 | 0 | 0 | 0 | 0.01 | 0.03 | 0 | 0.14 | 0.21 | 0 | 0.21 | 0.12 | 0.08 | 0.02 |
| **PHQ6** | 0 | 0 | 0 | 0.05 | 0.03 | 0 | 0 | 0.16 | 0.07 | 0.04 | 0.21 | 0 | 0.16 | 0.16 | 0.19 |
| **PHQ7** | 0 | 0 | 0 | 0.02 | 0 | 0.04 | 0.1 | 0.07 | 0.03 | 0.16 | 0.12 | 0.16 | 0 | 0.16 | 0 |
| **PHQ8** | 0 | 0 | 0 | 0 | 0.02 | 0.04 | 0.05 | 0.12 | 0.05 | 0.01 | 0.08 | 0.16 | 0.16 | 0 | 0.22 |
| **PHQ9** | 0 | 0.04 | 0.02 | 0 | 0.02 | 0 | 0 | 0.02 | 0.03 | 0 | 0.02 | 0.19 | 0 | 0.22 | 0 |


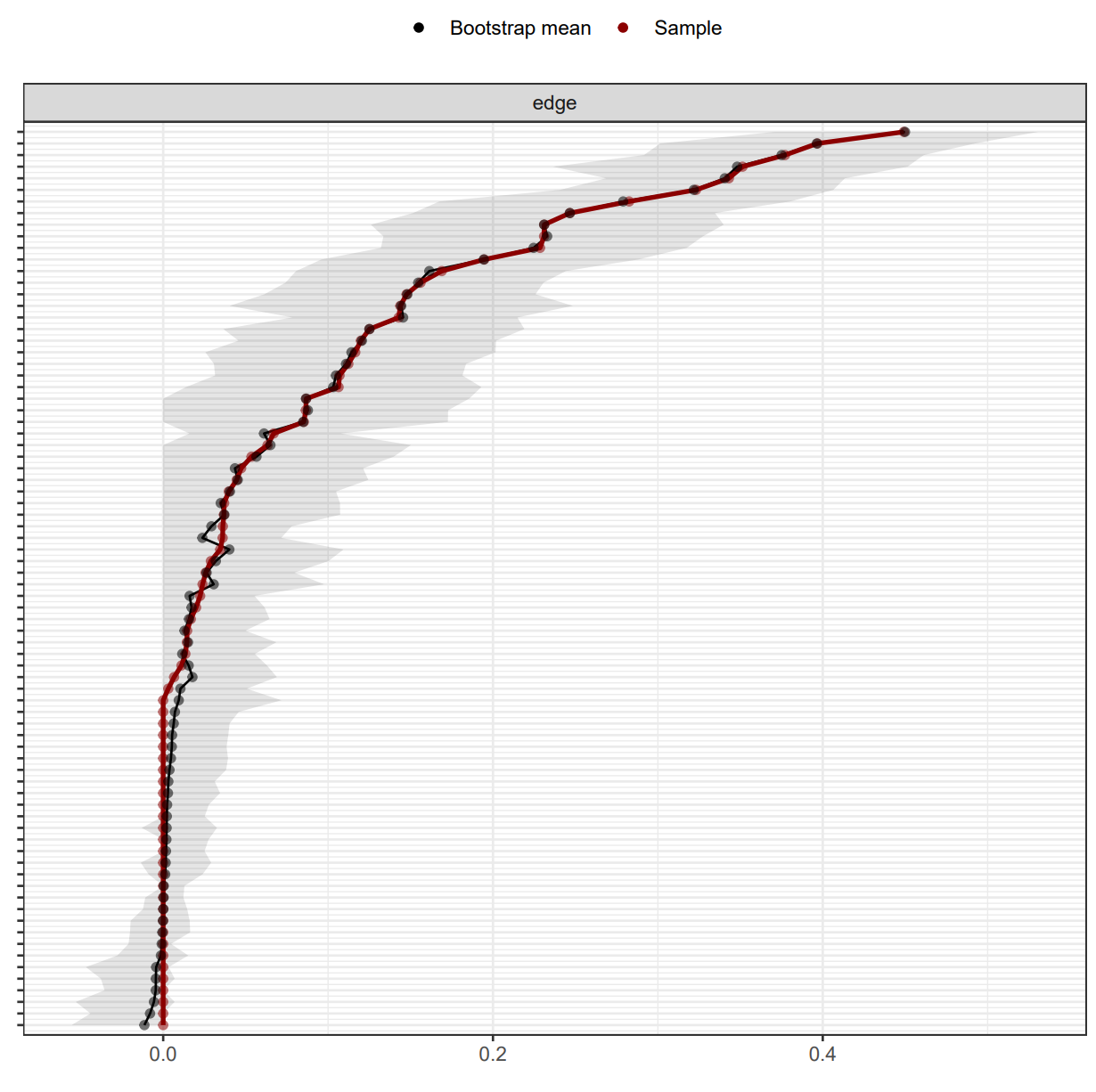


Figure S1. Accuracy of edge weights in the loneliness-anxiety network

*Note*: The red line depicts the sample edge weights and the gray bar depicts the bootstrapped confidence interval.


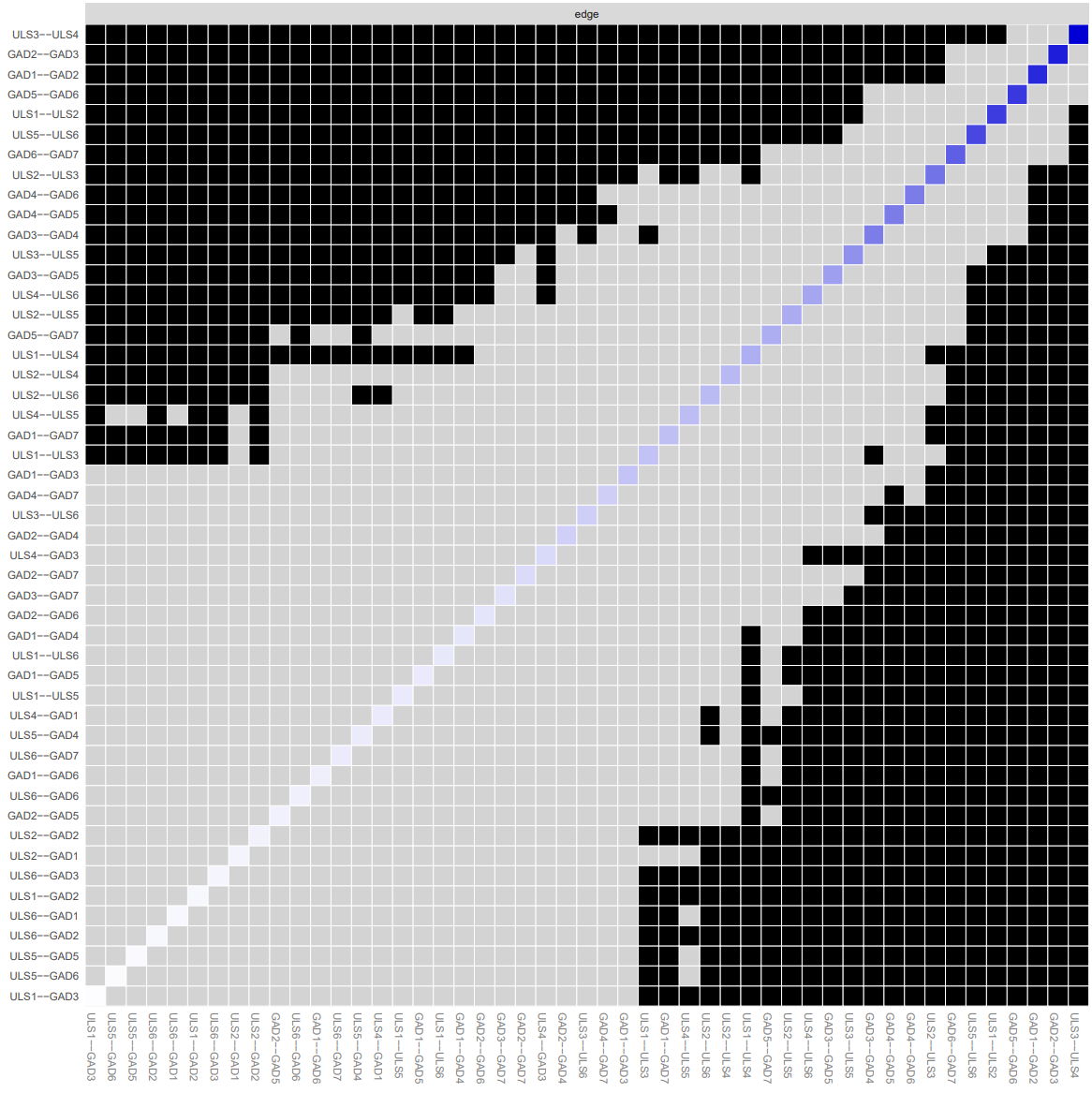


Figure S2. Bootstrapped difference test for edge weights in the loneliness-anxiety network

*Note*: Gray boxes indicate edge weights that do not differ significantly from one another, while black boxes indicate edge weights that do differ significantly. Blue and red boxes on the diagonal correspond to edge weights with positive and negative correlations, respectively.


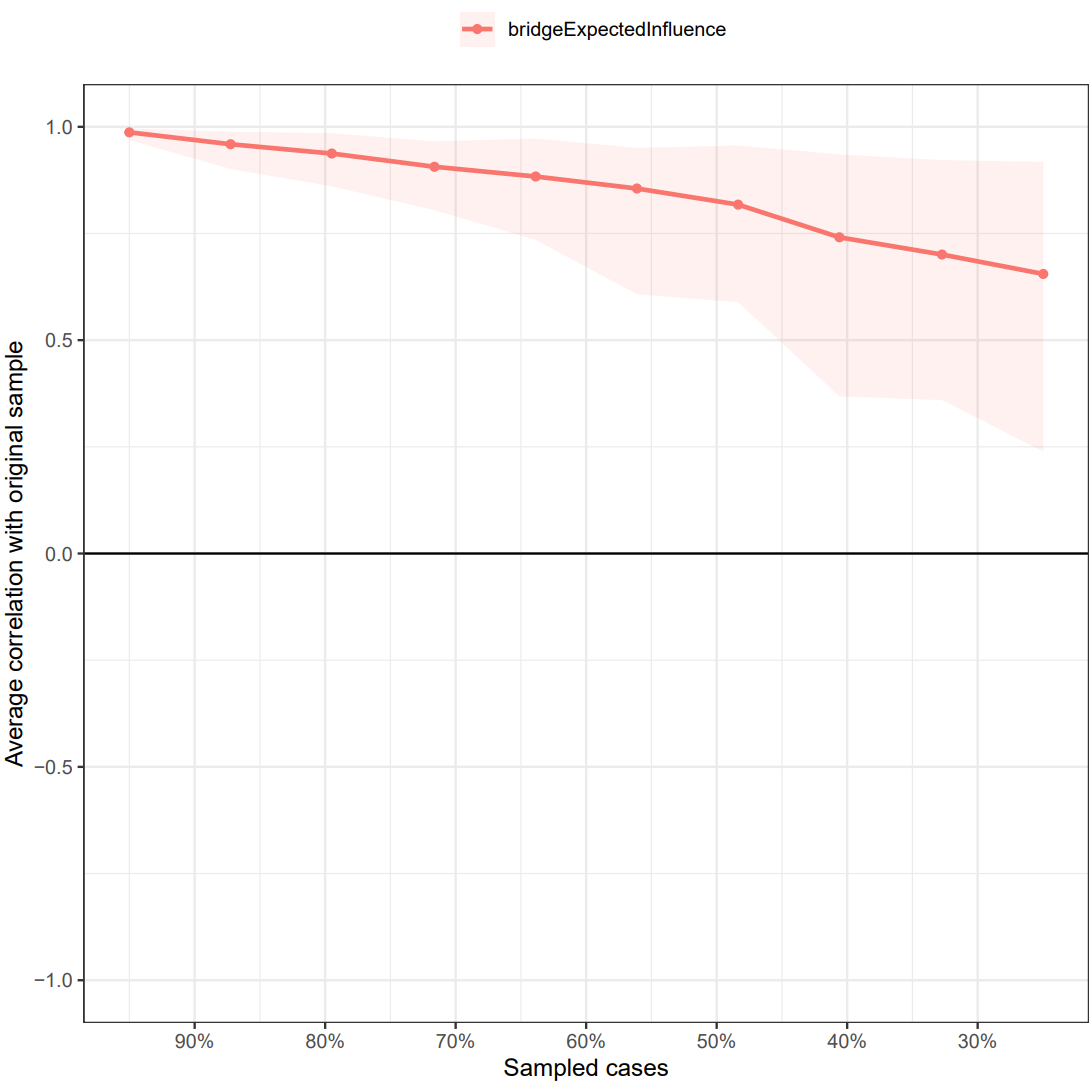


Figure S3. Stability of node bridge expected influences in the loneliness-anxiety network

*Note*: The red bar represents the average correlation between node bridge expected influences in the full sample and subsample with the red area depicting the 2.5th quantile to the 97.5th quantile.


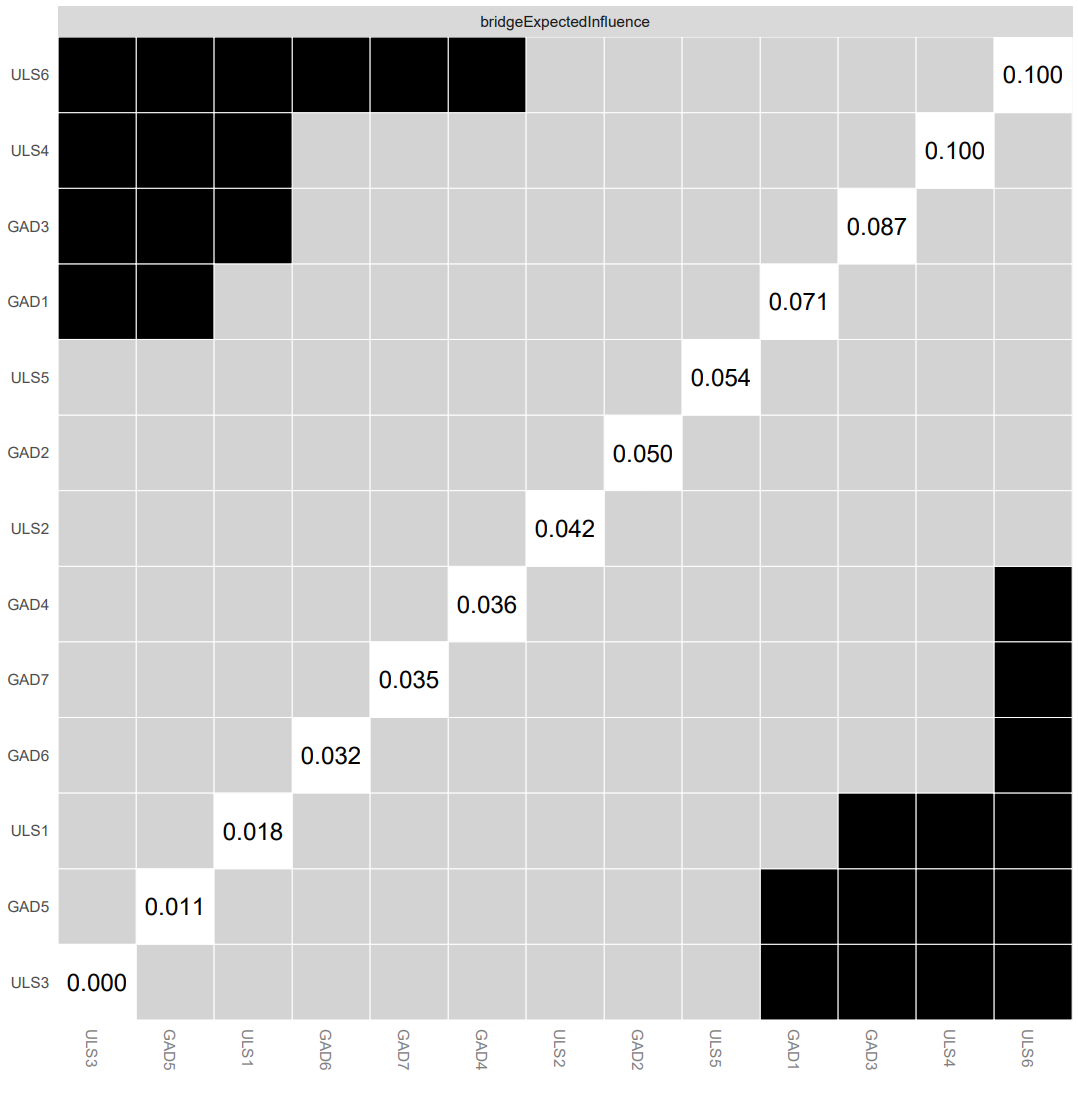


Figure S4. Bootstrapped difference test for node bridge expected influences in the loneliness-anxiety network

*Note*: Gray boxes indicate node bridge expected influences that do not differ significantly from one another, while black boxes indicate node bridge expected influences that do differ significantly.


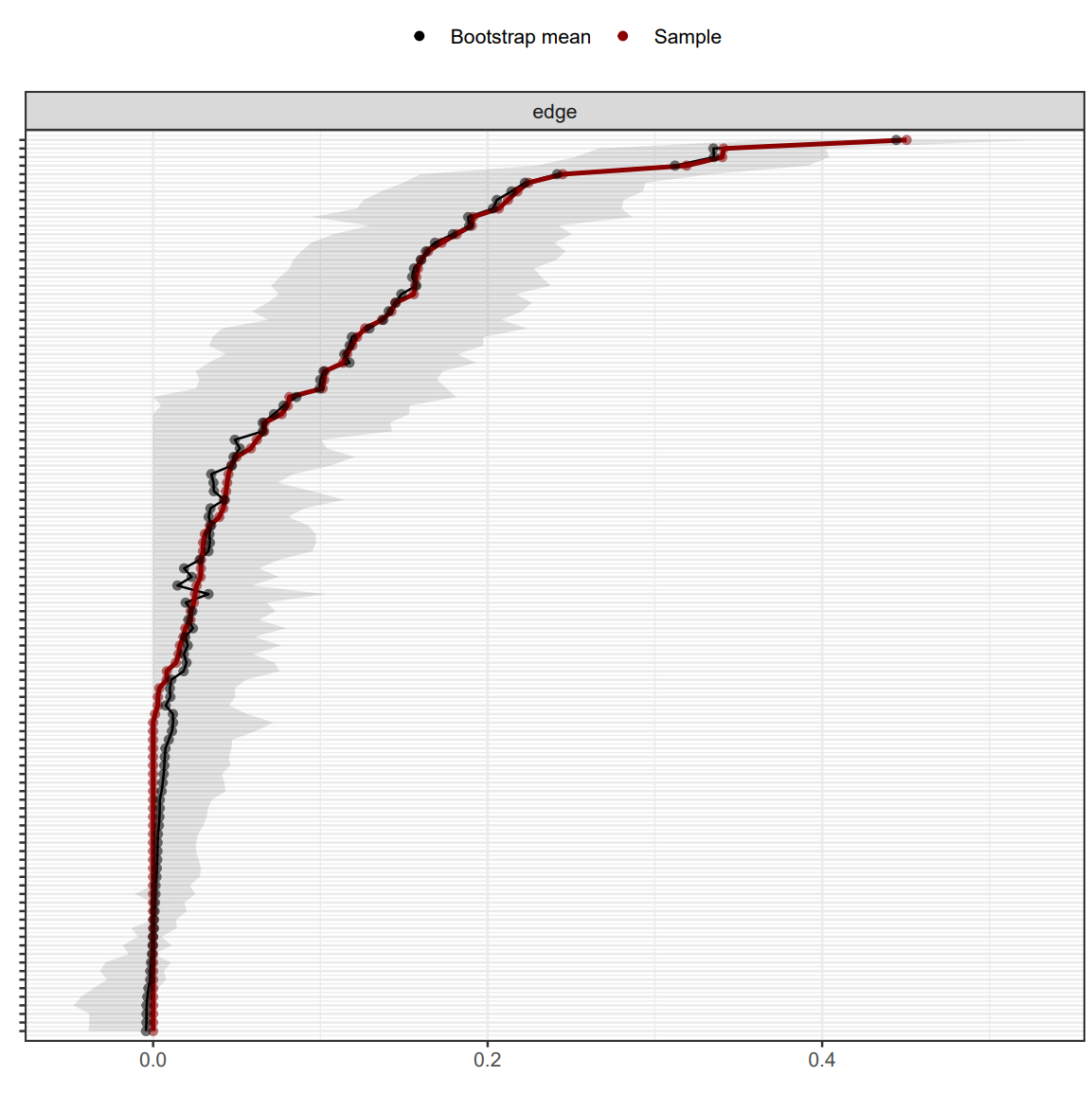


Figure S5. Accuracy of edge weights in the loneliness-depression network

*Note*: The red line depicts the sample edge weights and the gray bar depicts the bootstrapped confidence interval.


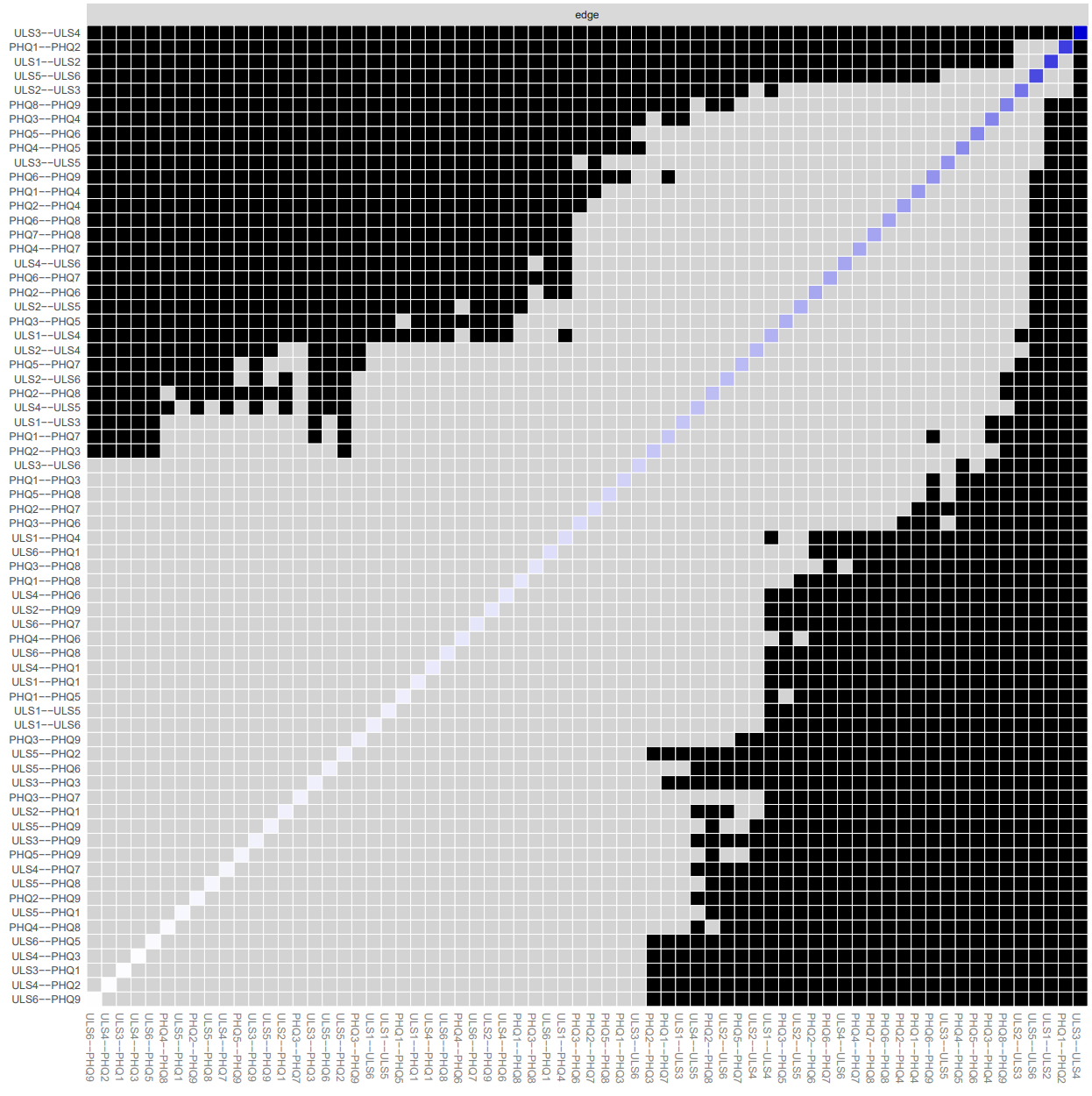


Figure S6. Bootstrapped difference test for edge weights within the loneliness- depression network

*Note*: Gray boxes indicate edge weights that do not differ significantly from one another, while black boxes indicate edge weights that do differ significantly. Blue and red boxes on the diagonal correspond to edge weights with positive and negative correlations, respectively.


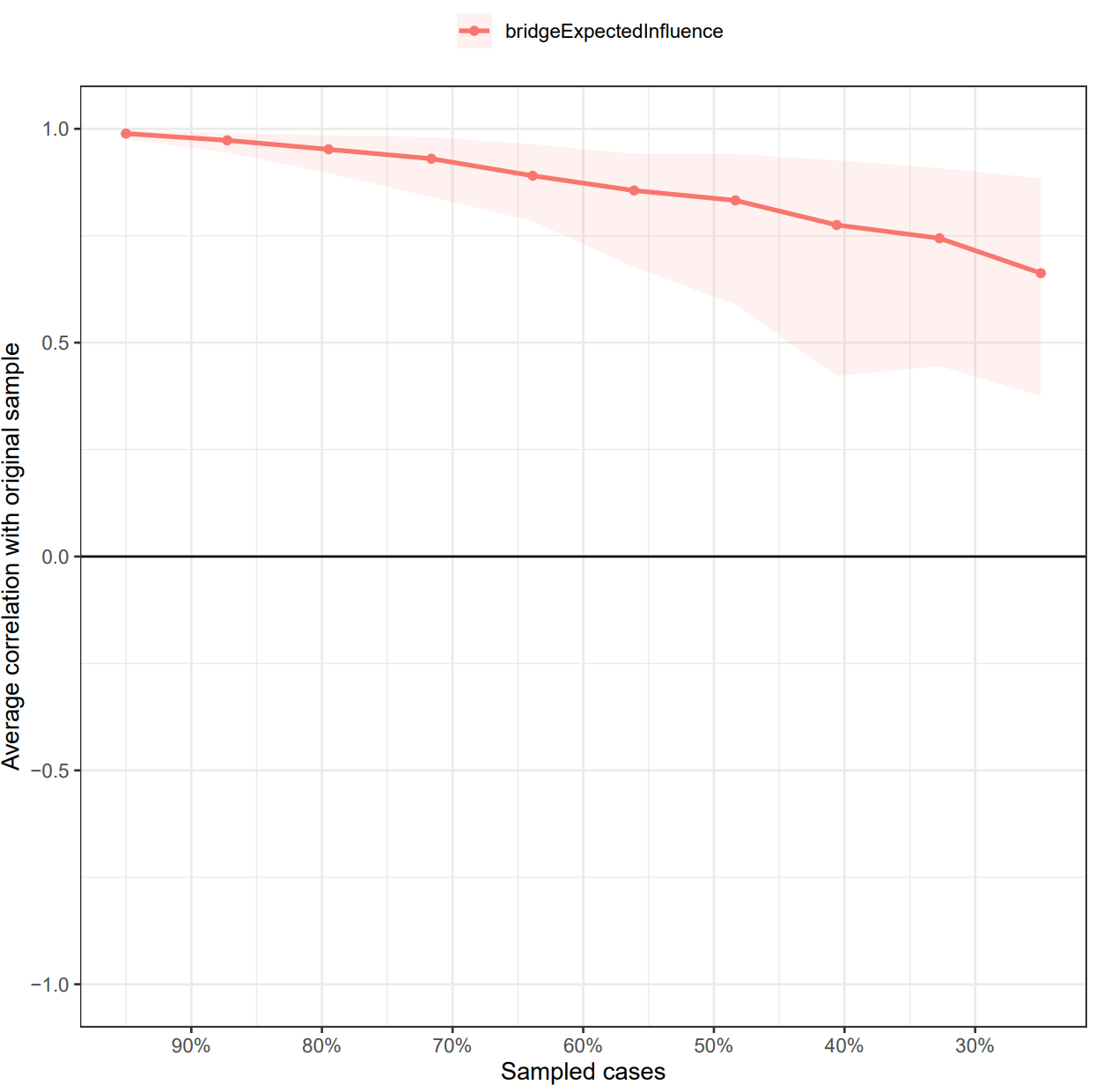


Figure S7. Stability of node bridge expected influences in the loneliness- depression network

*Note*: The red bar represents the average correlation between node bridge expected influences in the full sample and subsample with the red area depicting the 2.5th quantile to the 97.5th quantile.


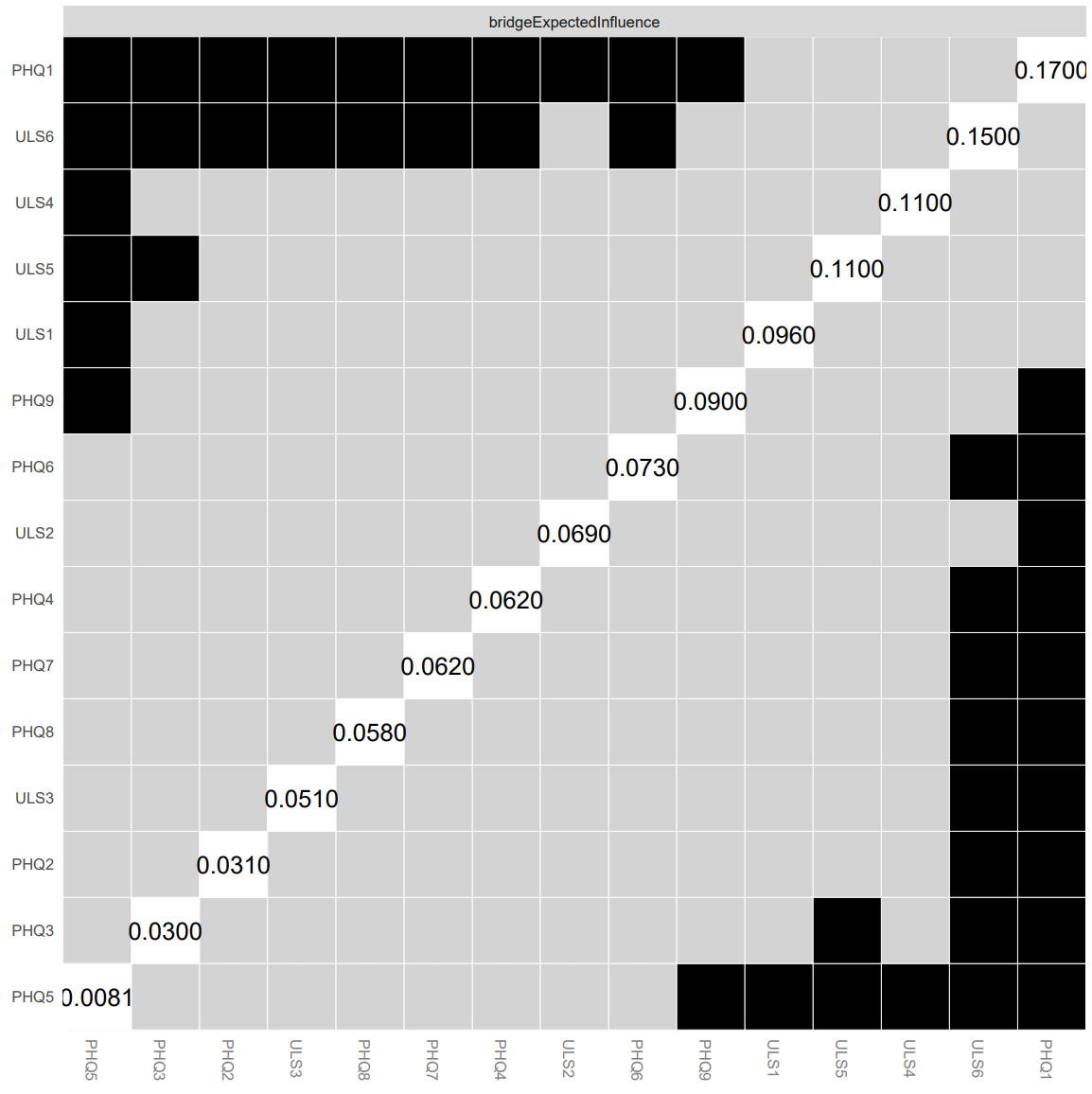


Figure S8. Bootstrapped difference test for node bridge expected influences in the loneliness- depression network

*Note*: Gray boxes indicate node bridge expected influences that do not differ significantly from one another, while black boxes indicate node bridge expected influences that do differ significantly.
