# Supplementary material for: How loneliness linked to anxiety and depression: a network analysis based on Chinese university students
Source: BMC Public Health. 2023 Dec 13;23:2499. doi: 10.1186/s12889-023-17435-4 (PMC10720215; doi:10.1186/s12889-023-17435-4)
Supplement: Supplementary file 2 — Supplementary Material 2 [file 12889_2023_17435_MOESM2_ESM.docx]

library(qgraph)

library(networktools)

library(ggplot2)

library(bootnet)

fulldata=read.csv("cleaned_itens.csv")

ULSitems <- fulldata[,c(1:6)]

PHQitems <- fulldata[,c(7:15)]

GADitems <- fulldata[,c(16:22)]

colnames(ULSitems)<-c("Companion","Seek\nhelp","Left\nout","Isolation","Unhappy","With\nme")

colnames(PHQitems)<-c("Anhe\ndonia","Sad\nmood","Sleep","Fatigue","Appetite","Worthless","Concen\n-tration","Motor","Suicide")

colnames(GADitems)<-c("Nervous","Control\nworry","Too\nmuch\nworry","Relax","Restless","Irritable","Afraid")

ULS_GADitems <- cbind(ULSitems, GADitems)

ULS_PHQitems <- cbind(ULSitems, PHQitems)

##The Loneliness-anxiety network

feature_group1 <- list("Loneliness"=(1:6), "Anxiety"=c(7:13))

mynetwork1 <- estimateNetwork(ULS_GADitems, default="EBICglasso", tuning=0.5,corMethod="cor",corArgs=list(method="spearman",use="pairwise.complete.obs"))

pdf("ULS_GADnetwork.pdf",width = 6,height = 6)

ULS_GADnetwork <- plot(mynetwork1, layout = "spring",

posCol=c("#150076"),negCol=c("#E4334B"),cut = 0.01,

color=c("#E58F7F","#E58F7F","#E58F7F","#E58F7F","#E58F7F","#E58F7F",

"#D696CA","#D696CA","#D696CA","#D696CA","#D696CA","#D696CA","#D696CA"),

legend.cex=0.6, legend.mode="groups",esize=14,

borders=T,border.width=3,border.color="black",mar=c(4,4,4,4),

groups=feature_group1,legend=F)

dev.off()

##Edge Weight accuracy and difference test

boot1 <- bootnet(mynetwork1, boots=2000,nCores=4,statistics=c("edge"))

#Accuracy of edge weights

plot(boot1, labels = FALSE, order = "sample")

#Edge weights difference test

plot(boot1,"edge",plot="difference",onlyNonZero = TRUE,order="sample")

##Estimate Bridge Expected Influence for each node

mybridge1 <- bridge(ULS_GADnetwork,communities = c('1','1','1','1','1','1','2','2','2','2','2','2','2'), useCommunities="all", directed=NULL,nodes=NULL)

plot(mybridge1,order="value",include ="Bridge Expected Influence (1-step)")

##Bridge Expected Influence stability test

caseDroppingBoot1 <- bootnet(mynetwork1,boots=2000,type="case",

statistics = "bridgeExpectedInfluence",

communities = feature_group1)

#get stability coefficients

corStability(caseDroppingBoot1)

#plot Bridge Expected Influence stability

plot(caseDroppingBoot1, statistics="bridgeExpectedInfluence")

##Bridge Expected Influence difference test

nonParametricBoot1 <- bootnet(mynetwork1,boots=2000,type="nonparametric",

statistics = "bridgeExpectedInfluence",

communities = feature_group1)

#plot Bridge Expected Influence difference

plot(nonParametricBoot1, statistics = "bridgeExpectedInfluence",order="sample",labels = TRUE,plot="difference")

##The Loneliness-depression network

feature_group2 <- list("Loneliness"=(1:6), "Depression"=c(7:15))

mynetwork2 <- estimateNetwork(ULS_PHQitems, default="EBICglasso", tuning=0.5,corMethod="cor",corArgs=list(method="spearman",use="pairwise.complete.obs"))

pdf("ULS_PHQnetwork.pdf",width = 6,height = 6)

ULS_PHQnetwork <- plot(mynetwork2,layout = "spring",

posCol=c("#150076"),negCol=c("#E4334B"),cut = 0.01,

color=c("#E58F7F","#E58F7F","#E58F7F","#E58F7F","#E58F7F","#E58F7F", "#FADE71","#FADE71","#FADE71","#FADE71","#FADE71","#FADE71","#FADE71","#FADE71","#FADE71"),

legend.cex=0.6, legend.mode="groups",esize=14,

borders=T,border.width=3,border.color="black",mar=c(4,4,4,4),

groups=feature_group2, legend=F)

dev.off()

##Flow network of suicide thoughts

graph_suicide <- flow(ULS_PHQnetwork, from="Suicide",horizontal =T,mar=c(5,5,5,5))

##Edge Weight accuracy and difference test

boot2 <- bootnet(mynetwork2, boots=2000, nCores=4, statistics=c("edge"))

#Accuracy of edge weights

plot(boot2, labels = FALSE, order = "sample")

#Edge weights difference test

plot(boot2, "edge", plot="difference", onlyNonZero = TRUE, order="sample")

##Estimate Bridge Expected Influence for each node

mybridge2 <- bridge(ULS_PHQnetwork,communities = c('1','1','1','1','1','1','2','2','2','2','2','2','2','2','2'),

useCommunities="all", directed=NULL,nodes=NULL)

plot(mybridge2, order="value",include ="Bridge Expected Influence (1-step)")

##Bridge Expected Influence stability test

caseDroppingBoot2 <- bootnet(mynetwork2,boots=2000,type="case",

statistics = "bridgeExpectedInfluence",

communities = feature_group2)

#get stability coefficients

corStability(caseDroppingBoot2)

#plot Bridge Expected Influence stability

plot(caseDroppingBoot2, statistics="bridgeExpectedInfluence")

##Bridge Expected Influence difference test

nonParametricBoot2 <- bootnet(mynetwork2,boots=2000,type="nonparametric",

statistics = "bridgeExpectedInfluence",

communities = feature_group)

#plot Bridge Expected Influence difference

plot(nonParametricBoot2, statistics = "bridgeExpectedInfluence",order="sample",labels = TRUE,plot="difference")
